# The impact of COVID-19 on hospital admissions and emergency department visits: A population-based study

**DOI:** 10.1371/journal.pone.0252441

**Published:** 2021-06-01

**Authors:** Elissa Rennert-May, Jenine Leal, Nguyen Xuan Thanh, Eddy Lang, Shawn Dowling, Braden Manns, Tracy Wasylak, Paul E. Ronksley

**Affiliations:** 1 Department of Community Health Sciences, Cumming School of Medicine, University of Calgary, Calgary, Alberta, Canada; 2 Department of Microbiology, Immunology and Infectious Diseases, University of Calgary, Calgary, Alberta, Canada; 3 Department of Medicine, University of Calgary, Calgary, Alberta, Canada; 4 O’Brien Institute for Public Health, University of Calgary, Calgary, Alberta, Canada; 5 Snyder Institute for Chronic Diseases, University of Calgary, Calgary, Alberta, Canada; 6 Infection Prevention and Control, Alberta Health Services, Calgary, Alberta, Canada; 7 Strategic Clinical Networks, Alberta Health Services, Calgary, Alberta, Canada; 8 Department of Emergency Medicine, University of Calgary, Calgary, Alberta, Canada; 9 Libin Cardiovascular Institute, University of Calgary, Calgary, Alberta, Canada; University of Malta Faculty of Health Sciences, MALTA

## Abstract

**Background:**

As a result of the novel coronavirus disease 2019 (COVID-19), there have been widespread changes in healthcare access. We conducted a retrospective population-based study in Alberta, Canada (population 4.4 million), where there have been approximately 1550 hospital admissions for COVID-19, to determine the impact of COVID-19 on hospital admissions and emergency department (ED visits), following initiation of a public health emergency act on March 15, 2020.

**Methods:**

We used multivariable negative binomial regression models to compare daily numbers of medical/surgical hospital admissions via the ED between March 16-September 23, 2019 (pre COVID-19) and March 16-September 23, 2020 (post COVID-19 public health measures). We compared the most frequent diagnoses for hospital admissions pre/post COVID-19 public health measures. A similar analysis was completed for numbers of daily ED visits for any reason with a particular focus on ambulatory care sensitive conditions (ACSC).

**Findings:**

There was a significant reduction in both daily medical (incident rate ratio (IRR) 0.86, p<0.001) and surgical (IRR 0.82, p<0.001) admissions through the ED in Alberta post COVID-19 public health measures. There was a significant decline in daily ED visits (IRR 0.65, p<0.001) including ACSC (IRR 0.75, p<0.001). The most common medical/surgical diagnoses for hospital admissions did not vary substantially pre and post COVID-19 public health measures, though there was a significant reduction in admissions for chronic obstructive pulmonary disease and a significant increase in admissions for mental and behavioral disorders due to use of alcohol.

**Conclusions:**

Despite a relatively low volume of COVID-19 hospital admissions in Alberta, there was an extensive impact on our healthcare system with fewer admissions to hospital and ED visits. This work generates hypotheses around causes for reduced hospital admissions and ED visits which warrant further investigation. As most publicly funded health systems struggle with health-system capacity routinely, understanding how these reductions can be safely sustained will be critical.

## Introduction

Extensive changes to healthcare systems have been seen globally since the beginning of the coronavirus disease 2019 (COVID-19) outbreak and subsequent pandemic. What initially began as an outbreak of pneumonia cases in Wuhan, China in December 2019 quickly spread from country to country with the first North American case in the United States (US) in January 2020 [1]. Given the contagious nature of COVID-19, patients in many jurisdictions were required to isolate themselves, and health care providers have delayed appointments or moved large portions of their practice to virtual visits and telemedicine for those requiring more urgent follow-up [2].

In addition to a shift to virtual healthcare, COVID-19 also influenced hospital admissions and visits unrelated to COVID-19 itself. There is emerging evidence from other countries that there are variations in healthcare utilization patterns due to COVID-19. For example, studies from both Spain and Italy have shown a reduction in admissions and procedures related to myocardial infarction and acute coronary syndrome [3, 4]. A recent study from Thailand [5] demonstrated that during a national lockdown for COVID-19 there was a significant reduction in daily emergency department (ED) visits. However, rates of admission and intensive care unit admissions were increased suggesting a potential delay to presentation [5]. Similarly, another study from Melbourne, Australia determined that during times of COVID-19 restrictions there was a significant reduction in ED visits [6]. Changes in hospital admissions due to COVID-19 in Canada have still not been broadly or systemically explored.

Alberta is a Canadian province with a population of approximately 4.4 million people that is serviced by one health care system for hospital and specialty care–Alberta Health Services (AHS). As of November 16, 2020, Alberta has had approximately 1550 cases of COVID-19 requiring hospitalization over the past several months, and a total of approximately 36405 cases. In Alberta, a state of public health emergency due to COVID-19 was declared under the public health act on March 15, 2020. Elective surgeries were postponed, and in-person healthcare visits were discouraged except where urgent. The public health emergency act expired on June 15, 2020 and elective surgeries commenced again. However, given that COVID-19 is a fluctuating circumstance, many elective surgeries have continued to be delayed as COVID-19 cases increase and virtual care remains a large component for many outpatient practices. Despite these reductions in outpatient service and elective surgeries, patients would presumably still require inpatient management for their acute and chronic medical conditions requiring admission through the ED, and the impact of the emergency measures on overall hospital use is uncertain. Moreover, if outpatient health care access was adversely impacted, an increase in ED visits for ambulatory care sensitive conditions (ACSC) would be expected [7].

In order to better understand the impact of changes associated with the COVID-19 pandemic on overall hospital use and patient safety, we conducted a population-based study to determine the impact on hospital and ED visits, assess markers of patient safety, and explore changes in diagnoses contributing to hospitalizations.

## Methods

### Study design and data collection

In order to examine daily hospital admissions and ED visits, we used a retrospective population-based design examining the adult (≥18 years) population of Alberta, Canada prior to the COVID-19 pandemic (March 16- September 23, 2019), until the COVID-19 pandemic started and the public health emergency act was implemented (i.e. post public health measures)–March 16 to September 23, 2020. We included data from January 1, 2019-March 15, 2020 to account for any seasonal variation that may have occurred in ED visits and hospital admission rates. At the time of this study, data was only available until September 23, 2020.

AHS analytics provided the numbers of daily hospital admissions from the Discharge Abstract Database (DAD), which includes all inpatient encounters within the province and provides detailed information on discharges, clinical information, diagnoses and procedures [8]. We obtained these numbers by day, main patient service (surgical/medical), elective or urgent, most responsible diagnosis, age group (18–64, 66–74, 75–84 and ≥85 years old), and hospital. We also collected information on all admissions through the ED for all acute care hospitals in Alberta and excluded admissions that occurred directly to a hospital ward, and those categorized as newborn, stillborn, or donor.

The National Ambulatory Care Reporting System (NACRS) was utilized to explore rates of visits to the ED (which may or may not have resulted in admission). The most responsible diagnosis for admissions and visits extracted from DAD and NACRS were identified by International Statistical Classification of Diseases and Related Health Problems– *10*^*th*^ *revision* (ICD-10) codes which represent the diagnosis resulting in the most hospital resources consumed and/or largest contributor to hospital length of stay.

### Statistical analyses and outcomes

Patient characteristics and demographics pre and post COVID-19 public health measures were compared using the student’s t-test, stratified by medical and surgical hospital admissions. Patient characteristics included length of stay (LOS), in-hospital mortality, medical complexity as defined by the Charlson comorbidity index [9], and Resource Intensity Weight (RIW). RIW reflect the intensity of resources used by patients based on different diagnostic, surgical procedure and demographic characteristics [10].

The primary outcome of this study was changes in number of hospital admissions and ED visits pre and post COVID-19. Changes in numbers of hospital admissions, for any reason including COVID-19, were compared pre and post COVID-19 using multivariable negative binomial regression models controlling for age, sex, Charlson comorbidity index and seasonality. This modeling approach was used to account for overdispersion observed within the hospitalization data. Two different models were conducted. The first explored the outcome of hospital admissions per day (reported as an incident rate ratio (IRR) with corresponding 95% confidence intervals (CI)) comparing the time period of March 16-September 23, 2019 to March 16-September 23, 2020. The second explored the same outcome but expanded the pre COVID-19 timeframe (January 1, 2019-March 15, 2020) versus March 16-September 23, 2020 in order to account for seasonality. All models were also stratified by medical versus surgical admissions. A sensitivity analysis utilizing an interrupted time series approach [11] was used to compare daily hospital admission rates pre COVID-19 and post implementation of public health measures.

The number of ED visits per day for any reason were calculated and compared between March 16-September 23, 2019 and March16-September 23, 2020. Rates of Ambulatory Care Sensitive Conditions (ACSC) were also explored using the Canadian Institute for Health Information ACSC indicator algorithm [12]. This includes admissions for the following seven medical conditions: hypertension, diabetes, angina, asthma, chronic obstructive pulmonary disease (COPD), epilepsy, and heart failure/pulmonary edema. Multivariable negative binomial models were used to calculate overall and condition-specific IRRs using the same timeframes described above.

The top 10 most responsible diagnoses from March 16-September 23, 2019 for both surgical and medical admissions were compared to the top 10 most responsible diagnoses from March 16-September 23, 2020. A student’s t-test was used to determine if there were significant changes in the frequency of any of these diagnoses between the two time periods.

Significance for all tests was determined as a p-value <0.05. All analyses were performed using Stata 16 (StataCorp 2019, College Station, TX).

### Ethical considerations

This research was approved by the University of Calgary Health Research Ethics Board (REB 20–0805). Informed consent for this study was waived by the ethics board.

## Results

The characteristics of patients admitted to hospital in Alberta pre and post COVID-19 public health measures are reported in Table 1. For medical admissions post COVID-19 public health measures, the Charlson comorbidity index was slightly lower at 1.62 (vs. 1.74 pre COVID-19, p = 0.001), there was a shorter LOS of 7.76 days (vs. 9.91 days pre COVID-19, p<0.001) and there was increased in-hospital mortality of 5.77% (vs. 5.30% pre COVID-19, p<0.001). For surgical admissions, post COVID-19 public health measures there was a shorter LOS of 6.85 days (vs. 8.66 days pre COVID-19, p<0.001) and a significant decrease in in-hospital mortality of 1.62% (vs. 2.06% pre COVID-19).

**Table 1:** Characteristics of patients pre COVID-19 (March 16-September 23, 2019) compared to post COVID-19 public health measures (March 16-September 23, 2020).

Characteristics	March 16-Sep 23, 2019	March 16-Sep 23, 2020	p-value
**Medical Admissions**			
N	77,897	68,704	
Male N (%)	38,649 (49.62)	35,206 (51.24)	<0.001
Mean age at admission in years (SD)	62.71 (20.52)	61.10 (21.31)	<0.001
Mean Charlson Comorbidity Index (SD)	1.74 (2.32)	1.62 (2.26)	<0.001
Mean Hospital Length of stay in days (SD)	9.91 (19.99)	7.76 (11.99)	<0.001
Mean Resource Intensity Weight (SD)	1.51 (2.65)	1.30 (1.77)	<0.001
In-hospital mortality N (%)	4,126 (5.30)	3,963 (5.77)	<0.001
**Surgical Admissions**			
N	16,162	14,463	
Male N (%)	8,455 (52.31)	7,491 (51.79)	>0.05
Mean age at admission in years (SD)	54.10 (19.9)	53.60 (19.7)	<0.05
Mean Charlson Comorbidity Index (SD)	0.57 (1.38)	0.54 (1.39)	<0.05
Mean Hospital Length of stay in days (SD)	8.66 (18.42)	6.85 (10.94)	<0.001
Mean Resource Intensity Weight (SD)	2.10 (4.35)	1.75 (2.84)	<0.001
In-hospital mortality N (%)	333 (2.06)	236 (1.62)	<0.01

Abbreviations: SD (standard deviation).

**Fig 1:**
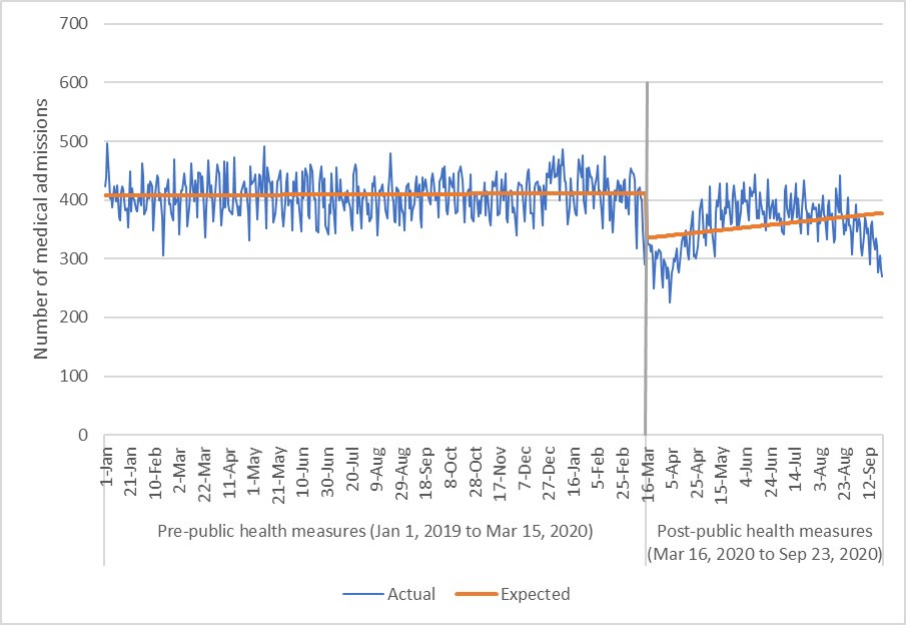
Number of medical hospital admissions via the emergency department per day.

**Fig 2:**
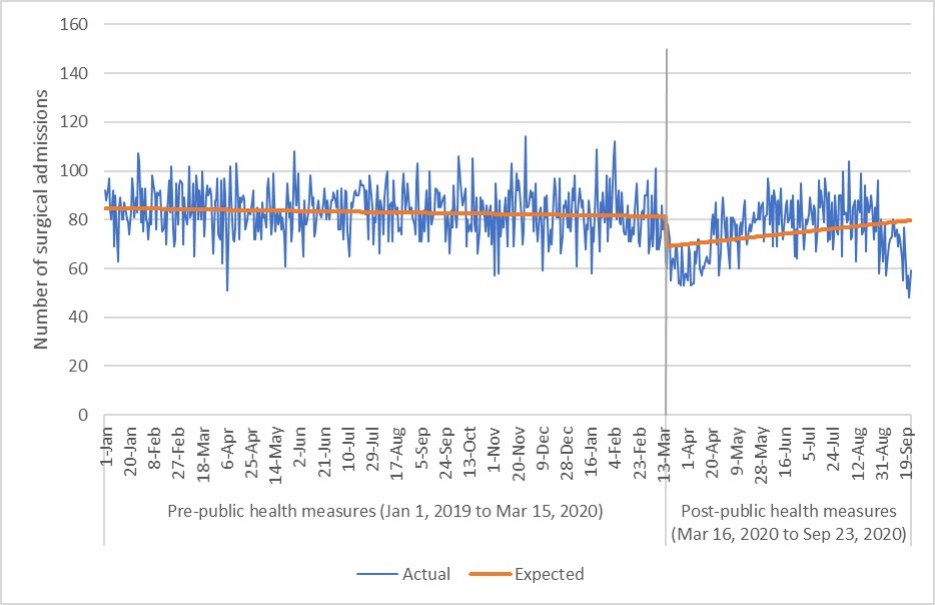
Number of surgical hospital admissions via the emergency department per day.

The number of medical and surgical admissions decreased significantly after implementation of public health measures March 16, 2020 (Figs 1 and 2). The negative binomial multivariable regression models comparing daily number of medical and surgical hospital admissions (Table 2) demonstrated that the COVID-19 period of March 16-September 23, 2020 was associated with a significant reduction in daily admissions with IRRs of 0.86 (95% CI 0.83,0.90, p<0.001) and 0.82 (95%CI 0.78,0.87, p<0.001), respectively. Our sensitivity analysis that accounted for potential seasonal effects showed a slightly smaller reduction in both medical (IRR 0.87, 95%CI 0.86,0.92, p<0.001) and surgical admissions (IRR 0.91, 95% CI 0.87,0.95, p<0.001) (S1 Table). The daily number of medical admissions was significantly higher in those with increasing age (IRR 1.01, 95%CI 1.00,1.02, p<0.05) and increasing Charlson comorbidity index (by increments of one) (IRR 1.11, 95%CI 1.02,1.21, p<0.05).

**Table 2:** Multivariable negative binomial regression with daily number of medical and surgical hospital admissions per day as the outcome comparing March 16-September 23, 2020 (post COVID-19 public health measures) to March 16-September 23, 2019.

		Negative binomial	
Covariates	IRR	95%CI	p-value
**Medical Admissions**			
Time period	0.10	0.10,0.10	<0.01
Post COVID-19 public health measures	0.86	0.83,0.90	<0.001
Sex—Male	0.50	0.35,0.73	<0.001
Age at admission	1.01	1.00,1.02	<0.05
Charlson Comorbidity Index	1.11	1.02,1.21	<0.05
**Surgical Admissions**			
Time period	0.10	0.10,1.00	<0.001
Post COVID-19 public health measures	0.82	0.78,0.87	<0.001
Sex—Male	1.11	0.87,1.42	>0.05
Age at admission	1.00	0.99,1.00	>0.05
Charlson Comorbidity Index	1.03	0.94,1.13	>0.05

Abbreviations: CI (confidence interval); IRR (incident rate ratio).

The interrupted time series analysis corroborated the findings of the negative binomial multivariable regression and demonstrated a reduction of approximately 11 surgical admissions per day (95%CI 7,16, p<0.001) and 69 medical admissions per day (95%CI 48,91, p<0.001) via the ED. This translates into a reduction of approximately 20% of admissions per day via the ED.

**Fig 3:**
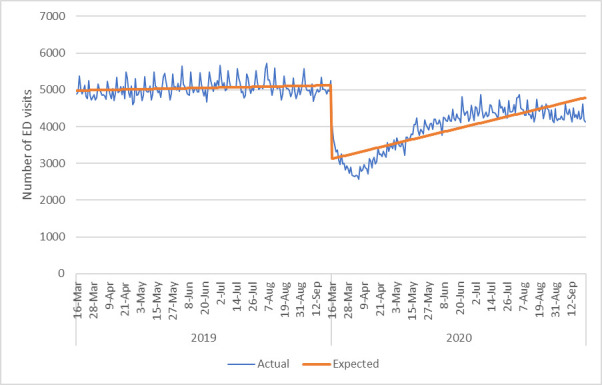
Number of emergency department visits per day for any reason.

**Fig 4:**
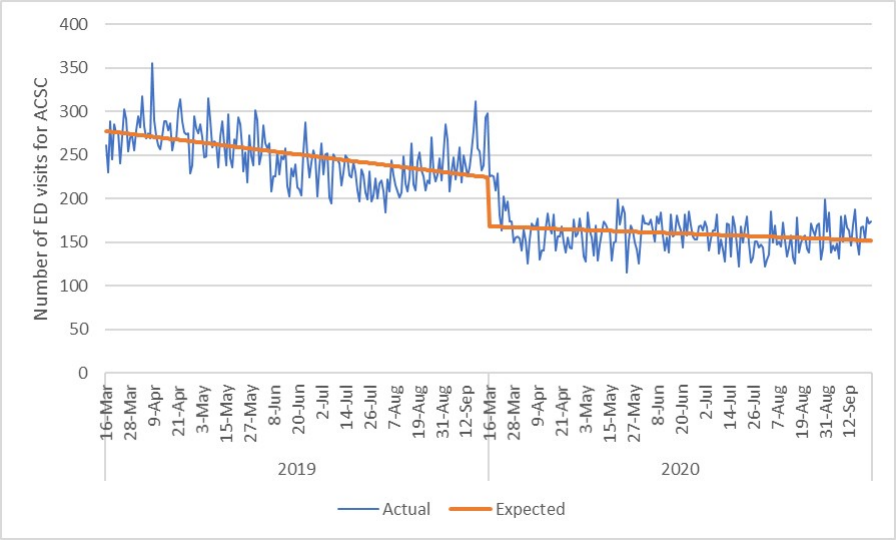
Number of emergency department visits per day for ambulatory care sensitive conditions.

The number of ED visits for any reason decreased significantly post COVID-19 public health measures (IRR 0.65, 95%CI 0.62,0.67, p<0.001) (Fig 3). The number of ED visits for ACSC also decreased significantly with the public health measures (IRR 0.75, 95%CI 0.72,0.79, p<0.001) (Fig 4) and did not appear to increase toward the end of the study period. When each ACSC was explored individually (Table 3), all decreased significantly following COVID-19 public health measures. There were significant reductions in ED visits for heart failure/pulmonary edema (IRR 0.83, p<0.001), COPD (IRR 0.75, p<0.001) diabetes (IRR 0.79, p<0.001) and hypertension (IRR 0.71, p<0.001).

**Table 3:** Multivariable negative binomial regression with daily number of ED visits for ACSC comparing March 16-September 23, 2020 (post COVID-19 public health measures) to March 16-September 23, 2019.

ACSC visits	IRR	95%CI	P-value
Angina	0.77	0.68,0.88	<0.001
Heart Failure/Pulmonary Edema	0.83	0.75,0.92	<0.001
Diabetes	0.79	0.70,0.88	<0.001
Asthma	0.85	0.76,0.96	<0.05
COPD	0.75	0.71,0.80	<0.001
Epilepsy	0.71	0.63,0.81	<0.001
Hypertension	0.71	0.65,0.79	<0.001

Abbreviations: ACSC (Ambulatory care sensitive conditions); IRR (Incident rate ratio); CI (Confidence interval).

The top most responsible diagnoses for hospital admissions post COVID-19 public health measures (i.e. March 16-September 23, 2020) were very similar to top diagnoses pre COVID-19 (i.e. March 16-September 23, 2019) which is demonstrated in Table 4. COPD and heart failure were still the two most common medical diagnoses for hospital admissions. However, the proportion of overall admissions accounted for by COPD did decrease significantly in the post COVID-19 public health measures period, from 5.11% to 3.48% (p<0.001).

**Table 4:** Top diagnoses March 16-September 23, 2020 (post COVID-19 public health measures) compared to top diagnoses March 16-September 23, 2019.

Medical Admissions								
March 16-September 23, 2019				March 16-September 23, 2020				p-value
Rank	ICD-10	Description	%	Rank	ICD-10	Description	%	
1	J44	Other chronic obstructive pulmonary disease	5.11	1	I50	Heart failure	3.78	>0.05
2	I50	Heart failure	3.98	2	J44	Other chronic obstructive pulmonary disease	3.48	<0.001
3	J18	Pneumonia, organism unspecified	3.09	3	F10	Mental and behavioral disorders due to use of alcohol	3.46	<0.001
4	I21	Acute myocardial infarction	2.80	4	I21	Acute myocardial infarction	2.61	<0.05
5	F10	Mental and behavioral disorders due to use of alcohol	2.65	5	I63	Cerebral infarction	2.25	>0.05
6	N39	Other disorders of the urinary system	2.34	6	J18	Pneumonia, organism unspecified	2.25	<0.001
7	I63	Cerebral infarction	2.23	7	N39	Other disorders of the urinary system	2.12	<0.01
8	Z51	Other medical care	1.78	8	Z51	Other medical care	1.82	>0.05
9	K85	Acute pancreatitis	1.54	9	K85	Acute pancreatitis	1.75	<0.01
10	L03	Cellulitis	1.48	10	L03	Cellulitis	1.68	<0.01
Surgical Admissions								
1	K35	Acute appendicitis	13.10	1	K35	Acute appendicitis	13.03	>0.05
2	K80	Cholelithiasis	9.21	2	K80	Cholelithiasis	10.14	<0.01
3	S72	Fracture of femur	9.03	3	S72	Fracture of femur	9.33	>0.05
4	S82	Fracture of lower leg	6.56	4	S82	Fracture of lower leg	6.78	>0.05
5	K56	Paralytic ileus and intestinal obstruction without hernia	3.82	5	K56	Paralytic ileus and intestinal obstruction without hernia	4.00	>0.05
6	T81	Complications of procedures not elsewhere classified	3.09	6	N13	Obstructive and reflux uropathy	3.08	>0.05
7	N13	Obstructive and reflux uropathy	2.75	7	S52	Fracture of forearm	2.93	<0.01
8	S52	Fracture of forearm	2.41	8	T81	Complications of procedures not elsewhere classified	2.72	>0.05
9	K81	Cholecystitis	2.14	9	K81	Cholecystitis	2.56	<0.05
10	K61	Abscess of anal and rectal regions	1.81	10	N20	Calculus of kidney and ureter	1.87	<0.05
12	N20	Calculus of kidney and ureter	1.56	12	K61	Abscess of anal and rectal regions	1.57	>0.05

## Discussion

Our work demonstrated a significant reduction in both daily medical and surgical hospital admissions following the period of COVID-19 (March 16-September 23, 2020). There was also a significant decline in visits to the ED for any reason as well as for ACSC specifically. The overall ten most common medical and surgical diagnoses for hospital admissions did not substantially change prior to COVID-19 and post COVID-19 public health measures. The proportion of total admissions accounted for by certain medical conditions did change, with COPD being significantly reduced but mental and behavioral disorders due to use of alcohol, and acute pancreatitis (which may be related to alcohol use), significantly increased. Surgical admissions were generally not significantly reduced though certain surgical admissions such as cholecystitis and forearm fracture significantly increased.

We determined that post COVID-19 public health measures there was a statistically significant decrease in the medical complexity of admitted patients (as defined by the Charlson Comorbidity Index). However, the change in the Charlson Comorbidity Index was small for both medical and surgical admissions and is unlikely to reflect a substantial difference clinically in patient comorbidities. Hospital LOS did decrease by approximately two days for both medical and surgical admissions, which is expected given pressure to reduce LOS in order to reduce patient exposure to COVID-19 in the hospital and retain bed capacity. The RIW decreased post COVID-19 public health measures which may reflect fewer tests and procedures being done given restricted access. Since we did not observe an increase in hospitalizations during the study period, the shorter LOS and fewer tests may have been appropriate, though longer-term analyses are required.

There have been an increasing number of publications available globally on changes in overall admissions due to health system changes required due to the COVID-19 pandemic [5, 6]. Other work done on specific medical conditions has demonstrated a reduction in acute coronary syndrome related admissions in several centers in northern Italy during COVID-19 [4]. Data from this time has suggested an increase in mortality that was not explained completely by COVID-19, and implied that perhaps patients did not seek medical attention when it was required [4]. While we did not assess mortality outside of the hospital environment, we did find a small increase in in-hospital mortality in the medical hospitalizations group, though the reasons for this are unclear. Given patient concerns with presenting to hospital during a pandemic and a decrease in access to outpatient testing and support, some patients who potentially had otherwise treatable conditions may have presented to care too late. Additionally, with restrictions on transfers during the COVID-19 period, patients that under optimal conditions would have gone from acute care facilities to hospice or palliative care would have stayed in hospital. There were no issues with reduced capacity in intensive care units in Alberta at the time of this study so the increased mortality would not have been a function of constrained critical care resources.

Another study also examining volume of visits to the ED found a significant reduction once the COVID-19 pandemic began [13]. They found that the delay to care resulted in increased numbers of cardiac arrests and worse outcomes for stroke patients [13]. While our study did not observe any increase in these conditions being hospitalized or presenting to the ED, it is possible that there will be worse patient outcomes in the long-term due to delayed care.

In Alberta, we observed substantial reductions in hospital admissions through the emergency department for both medical and surgical issues. While the top diagnoses seen in the ED were similar both pre and post COVID-19, there were significantly fewer medical admissions during COVID-19 for exacerbation of COPD and pneumonia of unspecified organism specifically. It is possible that enhanced personal hygiene practices and social distancing resulted in fewer viral respiratory infections, which are a known trigger for such medical conditions [14, 15]. Although there was a clear reduction in health care access, we did not note an increase in ED visits for ACSC, perhaps in part because these include COPD, which as noted above, were reduced.

Our work did demonstrate an increase in medical admissions due to mental and behavioral disorders due to use of alcohol and pancreatitis which can be triggered by alcohol consumption. A recent survey study demonstrated that there was an increase in adult alcohol consumption during the COVID-19 pandemic [16]. Alcohol consumption is tied to exacerbation of existing mental health issues or even triggering them [17]. These factors may explain the increase in admission seen due to these conditions.

Other potential explanations for the overall reduction in admissions, include the emotional toll of COVID-19 and the fear associated with it, and with seeking medical care [13, 18], and changes in healthcare provider behavior. During times of infectious diseases outbreaks and pandemics, there may be changes to physician behavior and clinical practice for various reasons including an understanding of limited resources resulting in more judicious use of admissions and tests, and also concerns for their own safety and safety of family members [19]. This combination of factors may partially explain some of our results. The lack of patient influx despite limited access to outpatient treatment could suggest the possibility that the medical system is over-treating patients who are able to manage without interventions or care, however, given the increase in in-hospital mortality and the short timeframe of the study we do not have enough evidence at this point to definitively make any conclusions around this.

Our work has several strengths. As mentioned above, previous studies have explored the impact of COVID-19 on certain types of admissions such as cardiac [3, 4], or in single centers [13]. This work is the first of its kind, of which we are aware, that explores a shift in admissions during the COVID-19 pandemic using a population-based approach, including possible reasons for this change. This work provides important insights into changes in healthcare utilization that occur during a pandemic, which are valuable nationally and globally. Our findings can potentially be used to plan interventions that relate not only to surge planning for hospitals during a pandemic but also pertain to hospital admissions generally, and whether certain conditions can be managed as outpatients or with reduced use of inpatient care. Understanding how to safely reduce healthcare is critical in systems that are generally overloaded with constrained resources.

Our study should be interpreted considering its limitations. Data were only available for March to September 2020, and we are currently not able to assess long-term impacts on the healthcare system due to COVID-19. It is possible that moving forward admission rates will increase and there will be negative outcomes from a lack of earlier interventions and medical care. In fact, it can be seen from Figs 1–3 that after an initial drop in visits to the ED for medical/surgical admissions and for any reason, these numbers of admissions and visits started to increase again. However, by September 2020 the graph demonstrates a decrease in numbers again which may have to do with rising COVID-19 cases in Alberta. This suggest that perhaps patients are avoiding the ED when community cases of COVID-19 rise due to concerns over transmission in hospital. Future data would need to be explored to provide a more conclusive explanation however. Secondly, mortality data outside of in-hospital rates were not available at the time of this work and so could not be used to understand if a lack of admissions to hospital was correlated with increased mortality outside of the hospital environment. Additionally, as this analysis only compares the two time points of 2019 to 2020, it is possible that there was an unrecognized underlying trend that may explain our results. This concern is somewhat mitigated by our additional analyses that expanded the pre COVID-19 timeframe from January 1, 2019-March 15, 2020 and compared it to March 16-September 23, 2020 (as opposed to the comparison of March 16-September 23, 2019 to March 16-September 23, 2020). Future work could consider a longer time period for an additional time series analysis. Finally, we did not assess ambulatory care utilization which could be explored through physician claims and outpatient databases to confirm if there was a shift in patients presenting to family physicians and specialists as outpatients.

Our work demonstrated that in a population that has relatively few cases of COVID-19 compared to other jurisdictions globally [20], there was still a profound impact on the healthcare system with reduced medical and surgical admissions and ED visits. The cause of this remains unclear but may include reduced viral respiratory infections overall (due to social distancing and improved hand hygiene) that may have previously worsened pre-existing conditions, changes in physician behaviour, and patients’ fear of presenting to acute care. This work provides an important foundation for further research around pandemic preparedness and planning. It has generated several hypotheses around causes for reduced hospital admissions and ED visits which can be utilized to target further research and investigation. Understanding how pandemics impact our health system capacity and delivery of health services is critical to creating strong health policy and ensuring quality safe care for patients.

## Supporting information

S1 Table Multivariable negative binomial regression with daily number of medical and surgical hospital admissions per day as the outcome comparing March 16-Sep 23, 2020 (post COVID-19 public health measures) to January 1, 2019-March 15, 2020. (DOCX)
