# Supplementary material for: The impact of COVID-19 on hospital admissions and emergency department visits: A population-based study
Source: PLoS One. 2021 Jun 1;16(6):e0252441. doi: 10.1371/journal.pone.0252441 (PMC8168854; doi:10.1371/journal.pone.0252441)
Supplement: S1 Table — (DOCX) [file pone.0252441.s001.docx]

S1 Table. Multivariable negative binomial regression with daily number of medical and surgical hospital admissions per day as the outcome comparing March 16-Sep 23, 2020 (post COVID-19 public health measures) to January 1, 2019-March 15, 2020.

| **Covariates** |  | **Negative binomial** | | |
| --- | --- | --- | --- | --- |
|  | **IRR** | | **95% CI** | **P-value** |
| **Medical Admissions** |  | |  |  |
| Time period | 1.00 | | 1,00,1.00 | >0.05 |
| Post COVID-19 public health measures | 0.87 | | 0.86,0.92 | <0.001 |
| Sex – Male | 0.66 | | 0.68,1.17 | <0.01 |
| Age at admissions | 1.01 | | 1.01,1.02 | <0.001 |
| Charlson Comorbidity Index | 1.05 | | 0.99,1.12 | >0.05 |
| Seasons: Fall reference | 1 | |  |  |
| Winter | 1.02 | | 0.99,1.04 | >0.05 |
| Spring | 0.99 | | 0.97,1.02 | >0.05 |
| Summer | 1.04 | | 1.02,1.07 | <0.001 |
| **Surgical Admissions** |  | |  |  |
| Time period | 1.00 | | 1.00,1.00 | >0.05 |
| Post COVID-19 public health measures | 0.91 | | 0.87,0.95 | <0.001 |
| Sex - Male | 1.15 | | 0.96,1.38 | >0.05 |
| Age at admissions | 1.00 | | 1.00,1.00 | >0.05 |
| Charlson Comorbidity Index | 1.03 | | 0.97,1.10 | >0.05 |
| Seasons: Fall reference | 1 | |  |  |
| Winter | 1.01 | | 0.98,1.05 | >0.05 |
| Spring | 0.98 | | 0.95,1.01 | >0.05 |
| Summer | 1.07 | | 1.04,1.10 | <0.001 |

Abbreviations: CI (confidence interval); IRR (incident rate ratio)
